# Investigation of Listening Effort in Tinnitus Patients by Providing Similar Peripheral Auditory Function With Control Group

**DOI:** 10.1002/brb3.70306

**Published:** 2025-02-16

**Authors:** Eser Sendesen, Meral Didem Turkyilmaz

**Affiliations:** ^1^ Department of Audiology Hacettepe University Ankara Turkey

**Keywords:** EEG alpha band, electroencephalography (EEG), extended high frequencies, listening effort, tinnitus

## Abstract

**Introduction:**

Previous studies have investigated listening effort in tinnitus patients compared to healthy individuals. These studies reported similar pure tone hearing thresholds between groups but did not investigate possible peripheral auditory dysfunction, which could affect the central auditory system and increase listening effort even when hearing thresholds are within the normal hearing range. This study aimed to investigate the presence of listening effort in tinnitus patients by controlling for peripheral auditory function (PAF).

**Methods:**

This study included 16 chronic tinnitus patients and 23 matched healthy controls, both with normal hearing thresholds. The subjects were assessed using 0.125–20 kHz pure‐tone audiometry, a visual analogue scale (VAS), the Montreal Cognitive Assessment (MoCA), the Tinnitus Handicap Inventory (THI), the matrix test, auditory brainstem response (ABR), and electroencephalography (EEG). EEG alpha band activity was recorded from parietal electrodes (P3, P4, P*z*).

**Results:**

The increase in alpha band power during the encoding phase of sentence presentation in tinnitus patients was less than that in the control group. We found higher VAS scores in tinnitus participants. We did not find significant differences in matrix test scores, ABR amplitude, or absolute latency values between groups. The EEG alpha power change and THI did not show a significant correlation.

**Conclusion:**

To the best of our knowledge, this is the first study to investigate the listening effort of tinnitus patients and healthy controls using EEG alpha band power while controlling for hearing and PAF. Tinnitus patients may expend more listening‐related effort despite having similar PAF to the control group.

## Graphical abstract

Topographical maps of EEG alpha band power during listening in Tinnitus and Control groups.

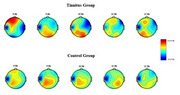

**Abbreviations**
CNS: central nervous system
EEG: electroencephalogram
MoCA: Montreal Cognitive Assessment test
THI: Tinnitus Handicap Inventory
VAS: visual analogue scale

## 1 Introduction

Tinnitus is a symptom that significantly affects people's daily lives (Rademaker et al. 2023). It is also known to impact various cognitive skills, such as attention and memory and may be associated with many semptomps like decresead sound tolerance (Waechter, Wilson, and Brännström 2021; Jensen et al. 2021; Altın et al. 2023). These cognitive skills have been shown to influence people's listening abilities in everyday situations, including listening effort (Francis and Love 2020; Degeest, Kestens, and Keppler 2022).

Listening effort refers to the allocation of cognitive resources to overcome the challenges encountered in maintaining a listening task (Pichora‐Fuller et al. 2016). Subjective, behavioral, and physiological methods are used to evaluate listening effort in the literature (Gürses et al. 2018; Alhanbali et al. 2019; Kılıç et al. 2024). Electroencephalography (EEG) and pupillometry are frequently used as physiological evaluation methods (Alhanbali et al. 2019; Giuliani et al. 2021). EEG provides insights into listening effort in the central nervous system, which arise from listening‐related changes in alpha band activity (Alhanbali et al. 2019; Seifi Ala 2022). However, pupillometry has been associated with the autonomic nervous system, where dilation in pupil response under adverse listening conditions has been interpreted as an indicator of listening‐related effort (Neagu et al. 2023).

Previous studies have demonstrated increased listening effort in tinnitus patients using objective (Sendesen and Turkyilmaz 2024; Sendesen et al. 2023; Sendesen and Türkyılmaz 2024; Callaway, Lunner, and Wendt 2018; Callaway, Lunner, and Wendt 2018; Cartocci et al. 2023) and subjective methods (Degeest, Kestens, and Keppler 2022; Huang et al. 2022; Shetty and Raju 2023; Degeest, Keppler, and Corthals 2017). Some studies used pupillometry to compare the listening effort of tinnitus patients and matched controls. Although they demonstrated that tinnitus patients experience listening difficulties, they reported that pupillometry may not provide reliable results because ANS activity in tinnitus patients differs from that in healthy individuals (Sendesen and Turkyilmaz 2024; Sendesen et al. 2023; Sendesen and Türkyılmaz 2024).

On the other hand, in these studies, the effect of hearing loss was controlled by ensuring similar pure tone hearing thresholds between the groups (Sendesen and Turkyilmaz 2024; Sendesen et al. 2023; Sendesen and Türkyılmaz 2024; Degeest, Keppler, and Corthals 2017). In a recent study, it was further ensured that hearing thresholds from 0.125 to 20 kHz were within normal limits (<20 dB HL) for both groups (Sendesen and Türkyılmaz 2024). However, previous work has shown that tinnitus patients may have peripheral auditory dysfunction (PAD) even if their hearing thresholds are normal (Kara et al. 2020; Ahmadpour et al. 2022; Sendesen et al. 2022).

Peripheral auditory function (PAF) is frequently evaluated in the literature using auditory brainstem response (ABR) and speech perception in noise (SPIN) tests (Kara et al. 2020; Sendesen et al. 2022; Monaghan et al. 2020; Çolak et al. 2024; Wilson and McArdle 2005). In ABR, PAD is associated with a decrease in Wave I amplitude, potentially due to cochlear synaptopathy, and an increase in Wave III and V amplitudes, which may indicate hyperactivity in the central auditory system (Bajin, Dahm, and Lin 2022). PAD may also be associated with an increase in the signal‐to‐noise ratio (SNR) in SPIN tests that reflect poor listening in noisy background (Kara et al. 2020; Çolak et al. 2024; Wilson and McArdle 2005; Bajin, Dahm, and Lin 2022; Plack et al. 2016).

Previous work have shown that PAD might cause cognitive problems as well (Monaghan et al. 2020; Kamerer et al. 2019; Pienkowski 2017). Furthermore, animal models have shown that PAD may directly cause listening difficulties (Henry 2022). Another study reported that PAD may cause listening difficulties in children with normal hearing (Dillon and Cameron 2021). This accumulating evidence may highlight the importance of controlling PAF in evaluating listening effort between the tinnitus and control groups, as subtle differences might be the root cause of increased listening effort in tinnitus patients. Here, we aimed to investigate the relationship between listening effort and tinnitus using EEG by ensuring similar PAF between groups and hearing loss.

## 2 Materials and Methods

### 2.1 Participants

The Non‐Interventional Clinical Research Ethics Committee provided ethical approval (GO22/1096) for this study. Written informed consent was obtained from the 54 participants. The tinnitus group consisted of tinnitus patients who applied to Hacettepe University Hospital. The control group was selected from campus volunteers who applied to our research announcement. As participation was voluntary, no participation compensation was paid to any participant.

Individuals who reported a pathology (such as otitis, demyelinating diseases, or cervical problems) associated with the etiology of tinnitus, those with a history of previous tinnitus therapy/treatment, those with acute tinnitus (less than 6 months), those with prolonged exposure to noise, or those taking antidepressants were excluded from the study based on the results of medical history, radiologic imaging, and audiologic evaluation. The inclusion criteria for both groups were that participants’ hearing thresholds between 0.125 and 20 kHz were within normal limits (<20 dB HL). Additionally, participants in the tinnitus group were required to have chronic tinnitus lasting more than 6 months.

The study included 42 participants who were native Turkish speakers and had similar levels of education (undergraduate or graduate). However, three participants, two from the control group and one from the tinnitus group, were excluded from the study due to high electrical artifact in EEG sessions. As a result, the tinnitus group included 16 participants (8 males, 8 females) aged 19–34. The healthy control group consisted of 23 participants (10 males, 13 females) aged 19–30 without tinnitus.

Participants had normal outer and middle ear functions in otoscopic and tympanometric exams. Pure tone audiometry (PTA) was performed using an Interacoustics AC‐40 audiometer, calibrated TDH‐39P headphones, a Sennheiser HDA200 (8–20 kHz), and a Radioear B‐71 bone vibrator. The Montreal Cognitive Assessment test (MoCA), a screening test that evaluates various domains of cognitive function, was administered to the participants. A score of 21 or higher indicates normal cognitive function (Dalrymple‐Alford et al. 2010). This test was used to ensure that participants could adequately understand and perform complex cognitive tasks, such as the tinnitus assessment process and the matrix test (MT), and to ensure consistency of responses to the questionnaire. Participants who were considered to have normal cognitive functioning based on the MoCA were included in the study. In the present study, we used the visual analogue scale (VAS) to subjectively assess listening effort, as no validated listening effort questionnaire was available in the participants’ native language. In the VAS assessment, 0 indicates “never happens” and 10 indicates “I always make an effort.”

### 2.2 Tinnitus Assessment

Tinnitus with frequencies below 8 kHz was assessed using TDH‐39P headphones, whereas tinnitus with frequencies above 8 kHz was assessed using Sennheiser HDA200 headphones. Tinnitus assessment started with TDH‐39P headphones. Sennheiser HDA200 headphones were used when necessary. Auditory stimuli were presented binaurally in tinnitus pitch matching. A two‐alternative forced selection procedure was used at 30 dB SL between 0.125 and 20 kHz (Basile et al. 2013; Tyler and Conrad‐Armes 1983). Subsequently, the tinnitus loudness level was matched in 5 dB increments, considering the ipsilateral hearing threshold of the participants at the tinnitus frequency (Tyler and Conrad‐Armes 1983).

The minimum masking level (MML) is the level at which tinnitus becomes inaudible. It is determined by incrementing the narrowband noise, whose center frequency matches the tinnitus frequency in 5 dB steps. Narrowband noise was presented binaurally, as all participants had bilateral/in the head tinnitus.

Residual inhibition (RI) is a phenomenon that examines the suppression of tinnitus after the presentation of an auditory stimulus. According to previous studies, RI can provide information about tinnitus characteristics, such as whether tinnitus is associated with increased spontaneous activity in the peripheral auditory system (Galazyuk et al. 2019). Therefore, to better understand the characteristics of tinnitus, we investigated the RI of the participants. RI was determined by presenting narrowband noise with the center frequency at the tinnitus frequency. This noise was presented 10 dB above the MML for 60 s (presented binaurally as all participants had bilateral/in the head tinnitus). These results were considered positive if the level of tinnitus perception decreased and negative if the level of tinnitus perception did not change or increased.

### 2.3 Tinnitus Questionnaire

Tinnitus Handicap Inventory (THI) was used to assess tinnitus discomfort level in participants’ daily lives (Aksoy, Firat, and Alpar 2007). THI consists of 25 questions designed to assess patients’ subjective psychological impacts of tinnitus. THI provides a tinnitus assessment including functional, emotional, and destructive subscales. “Yes,” “no,” and “sometimes” are the answers. The following answers are scored 4, 2, and 0 points, respectively.

### 2.4 Visual Analogue Scale

The VAS provided general information about the participants’ listening efforts. Participants were asked to mark a scale between 0 and 10 points containing the question “What is the frequency of your listening effort in daily life.” The term “listening effort” was explained to participants as “the effort made while listening in daily life.” 0 points means “it never happens,” and 10 points means “I make an effort all the time.”

### 2.5 Auditory Brainstem Responses

The Vivosonic Integrity ABR device and EAR‐TONE 3A insert headphones were used. The ABR protocol applied in this study was designed by taking previous studies as reference (Sendesen et al. 2022; Kim and Han 2023). Two channels of ABR were used with an electrode montage of the forehead (non‐inverting), low forehead (ground), and mastoids (inverting). The impedances for absolute electrodes were ≤5 kΩ and for inter‐electrodes were ≤2 kΩ. Participants were instructed to remain as still as possible to minimize EEG variability. Click stimuli of alternating polarity (to prevent amplitude variations in ABR waveforms resulting from rarefaction or condensation stimuli (Tietze and Pantev 1986)) at 80 dB nHL level (21.1 click/s, 2000 sweeps, 100 µs duration) were used. As all participants had binaural/in the head tinnitus, both ears were evaluated separately. The responses underwent bandpass filtration of 100–1500 Hz. The analysis time was 15 ms. The interpeak amplitude ratios III–I, V–III, and V–I, as well as the Wave I–V latencies and amplitudes of the subjects, were calculated.

### 2.6 Speech Reception Thresholds (SRT)

The MT (Zokoll et al. 2013) determined participants’ 50% SRT level and was also used as a SPIN test to assess PAF (Kara et al. 2020). The MT material contains 10 words from each vocabulary category (name, numeral, adjective, object, and verb). Despite having only 50 words in total, the possibility of freely combining words from different categories leads to 100,000 possible sentences. All words in the MT are commonly encountered in daily life. Participants’ chances of guessing were low because the sentences were syntactically correct but lacked semantic coherence. Thirty distinct lists, each comprising 20 sentences, were used in a random manner for each participant without any repetition. At the start of testing, sentences were presented in an open‐set format with Sennheiser HDA200 headphones at 0 dB SNR. The noise level (65 dB SPL) was kept constant, whereas the speech stimulus level was adaptive (changing in 1 dB SPL steps). The SNR level decreased proportionally if participants achieved more than 50% correct answers and increased proportionally if they fell below. As a result of the test, SNR levels were obtained at which 50% of the presented words were correctly identified. As all participants had bilateral or in‐the‐head tinnitus, both ears were evaluated separately.

MT (Zokoll et al. 2013) determined participants’ 50% SRT level and was also used as a SPIN test to assess PAF (Kara et al. 2020). The MT material contains 10 words from each vocabulary category (name + numeral + adjective + object + verb). Despite having only 50 words in total, the possibility to freely combine words between different word categories leads to 100,000 different possible sentences. All words in the MT are commonly encountered in daily life. Participants’ chances of guessing were low because the sentences were syntactically correct but lacked semantic coherence. Thirty distinct lists, each comprising 20 sentences, were employed in a random manner for each participant without any repetition. At the start of testing, sentences were presented using an open‐set presentation format with Sennheiser HDA200 headphones at 0 dB SNR. The noise level (65 dB SPL) was kept constant, whereas the speech stimulus level was adaptive (changing in 1 dB SPL steps). The SNR level decreases proportionally if participants achieved more than 50% correct answers and increased proportionally if they fell below. As a result of the test, SNR levels were obtained at which 50% of the presented words were correctly identified. As all participants had binaural/in the head tinnitus, both ears of the participants were evaluated separately.

### 2.7 Speech Materials and Background Noise

In the present study, we used the noise‐vocoded sentences previously used by Miles et al. to generate listening effort in participants (Miles et al. 2017). Multi‐talker babble noise (with 16 talkers) from multiple speakers in the background was combined with the MT's sentences using MATLAB scripts. Stimuli were divided into channels separated by 16 logarithmic intervals. The amplitude envelope was generated by extracting the absolute value of each channel from the Hilbert transform. The extracted envelope was used to modulate noise with the same frequency band. In a speech recognition task, Miles et al. (2017) found that parietal alpha band power decreased when noise vocoding changed signal spectral content and speech intelligibility (Miles et al. 2017). So, the background noise and noise‐vocoded sentences were generated by recombining each noise band. The background noise and root mean square levels of the sentences were equalized in MATLAB after the vocoding process. The cumulative duration of the stimulus is 6 s. It comprised noise from 0 to 1 s, noise‐vocoded speech from 1 to 4.5 s, and noise from 4.5 to 6 s. As all participants had binaural/in the head tinnitus, the stimuli were presented binaurally.

### 2.8 EEG Recording

EEG recordings were recorded during the speech recognition task in a soundproof room. The stimulus was presented as 6‐channel noise‐vocoded sentences at 50% SRT (6ch50% SRT) level determined in the MT. The stimulus lasted 9 min. Participants were instructed to repeat the sentence at noise offset. As the participants were in a different room from the researchers, a video camera and a voice recorder were used to ensure that participants were paying attention to the experiment. This was communicated to participants to help maintain their focus on the task.

Continuous EEG data were recorded with a 21‐channel NuAmps II Neuroscan amplifier. In the standard 10–20 configuration, 19 scalp electrodes were placed. Electrical activity was recorded from both earlobes. The common average reference was calculated using A2 as the reference electrode during analysis. During recording, all electrode impedances were below 5 kΩ. A notch filter removed 50 Hz artifact from EEG continuous data filtered between 1 and 60 Hz. EEGlab v14.1.2 was used a standard ocular reduction algorithm for ocular artifact rejection. EEG continuous data revealed the alpha band with an 8–12 Hz band pass filter. Hilbert transform was used for EEG segment envelopes. The parietal alpha band has been linked to speech and nonspeech tasks, so it is used in listening effort studies (Obleser and Weisz 2012; Wisniewski, Thompson, and Iyer 2017). On a trial‐by‐trial basis, the parietal electrodes (P3, P4, and P*z*) were used to extract the absolute value of the alpha band during the encoding period [1 s time interval 200 ms before the end of the noise‐vocoded speech (3.3–4.3 s)] and baseline in noise (300–800 ms after noise onset). For each trial, relative percent change was calculated as mean alpha band power during encoding minus mean alpha band power during baseline, divided by mean alpha band power during baseline. This was multiplied by 100 to report a percent change from baseline.

### 2.9 Statistical Analysis

The G*Power program was used to calculate the sample size for this study. On the basis of the mean and standard deviation values derived from the pilot study, it is expected that 14 participants from each group be included in this study. This is required to ensure a 5% type I error level and 95% power to detect a minimal, significant difference. The SPSS version 25.0 (IBM Inc., Armonk, NY, USA) package program was used to evaluate the data. All data had a normal distribution. Age, MoCA scores, EEG alpha band, and ABR results between groups were assessed using the independent sample *t*‐test. The assumption of homogeneity of variance was checked using Levene's test. Further analysis was conducted using Pearson's correlation coefficient to assess the association between the EEG alpha band and the THI outcomes. Gender differences between the groups were evaluated with Fisher exact test. The *p* value threshold for statistical significance was set at 0.05.

## 3 Results

### 3.1 Population

**FIGURE 1:**
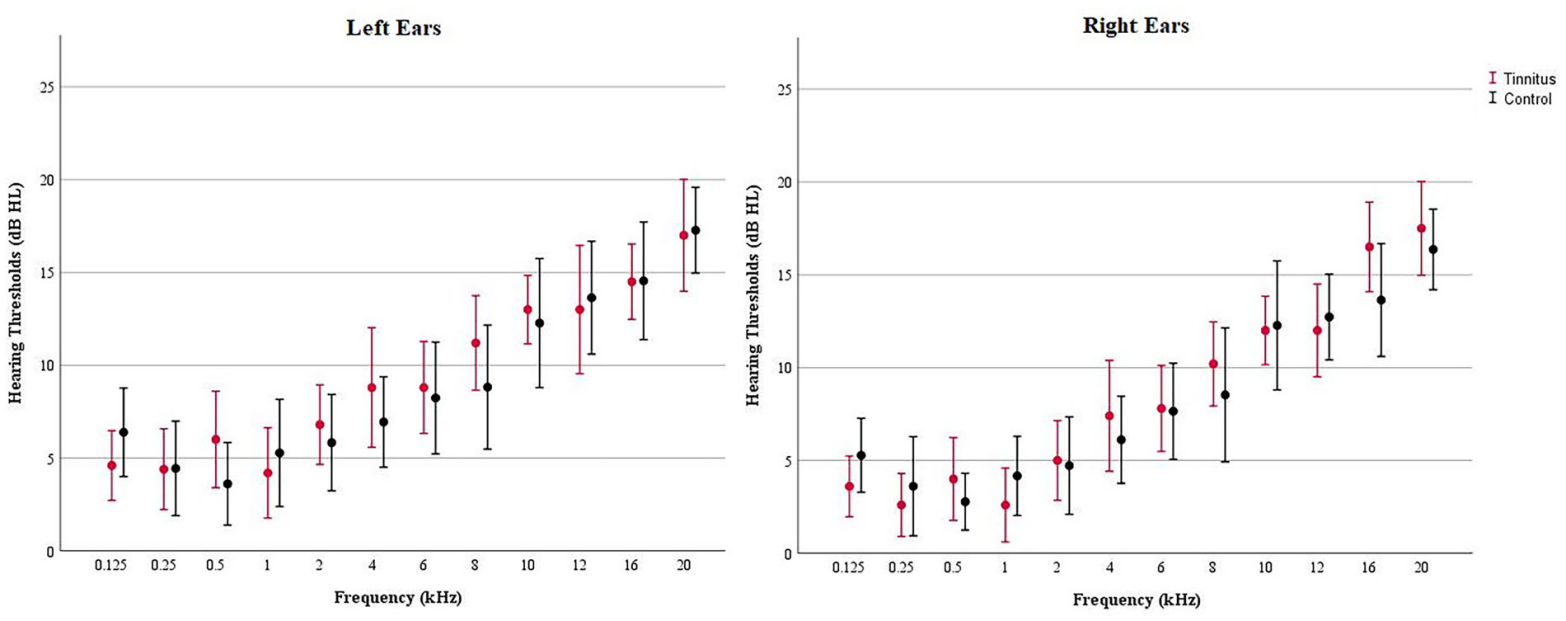
The mean hearing thresholds of the right and left ears according to groups.

All pure‐tone thresholds (PTTs) for each frequency (0.125–20 kHz) were not significant between groups (*p* > 0.05). The mean hearing thresholds are shown in Figure 1.

All participants had a MoCA score of more than 21. Participants’ demographics and tinnitus characteristics are shown in Tables 1 and 2, respectively.

**TABLE 1:** Demographic characteristics of the subjects.

	Tinnitus group	Control group	*p* value
**Age**	23 ± 3 (19–34)	22 ± 2 (19–30)	0.83
**Gender**			0.79
Male	7	10	
Female	9	13	
**MoCA**	29 ± 1 (27–30)	29 ± 1 (28–30)	0.93

Abbreviation: MoCA, Montreal Cognitive Assessment test.

**TABLE 2:** Tinnitus characteristics and Montreal Cognitive Assessment (MoCA) scores of the tinnitus group.

Subject no	Tinnitus location	Tinnitus pitch (kHz)	Tinnitus loudness level (dB)	Minimal masking level (dB)	Residual inhibition	THI score	MoCA score
1	Bilateral	14	35	10	+	42	29
2	Bilateral	8	45	25	+	62	28
3	In the head	10	30	25	+	38	29
4	Bilateral	8	30	25	+	36	30
5	Bilateral	6	35	30	+	30	30
6	In the head	6	40	20	+	62	28
7	Bilateral	4	50	35	−	30	30
8	In the head	6	35	25	+	56	29
9	In the head	10	70	30	+	50	30
10	In the head	6	45	30	−	48	30
11	In the head	10	80	30	+	68	29
12	Bilateral	0.25	35	15	+	24	28
13	In the head	0.25	30	25	−	60	30
14	In the head	18	10	0	+	90	28
15	Bilateral	6	30	20	−	14	27
16	In the head	8	55	35	+	86	29

Abbreviation: THI, Tinnitus Handicap Inventory.

### 3.2 Comparison of EEG Alpha Band Power Results Between Groups

**FIGURE 2:**
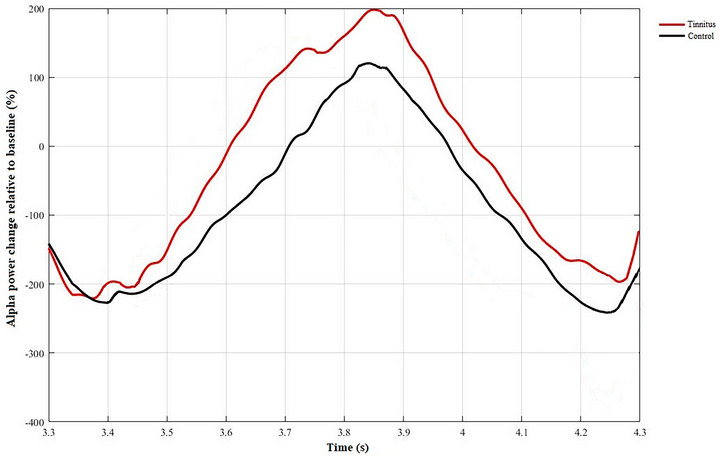
Time‐dependent change of EEG alpha band power based on parietal electrodes (P3, P4, P*z*). EEG, electroencephalography.

**FIGURE 3:**
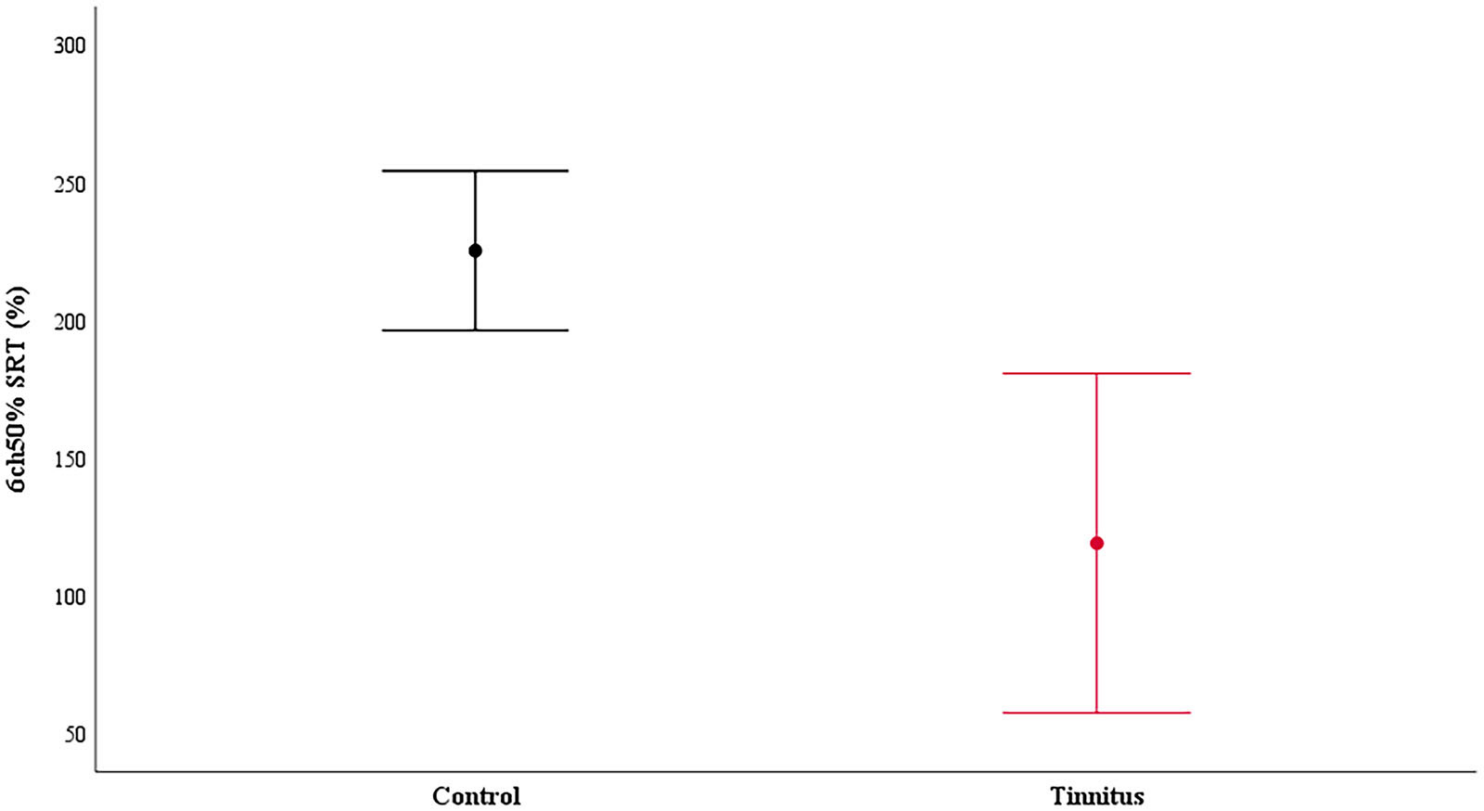
Mean EEG alpha power change differences between tinnitus (*n* = 16) and control (*n* = 23) groups shown with error bars. EEG, electroencephalography; SRT, speech reception thresholds.

**FIGURE 4:**
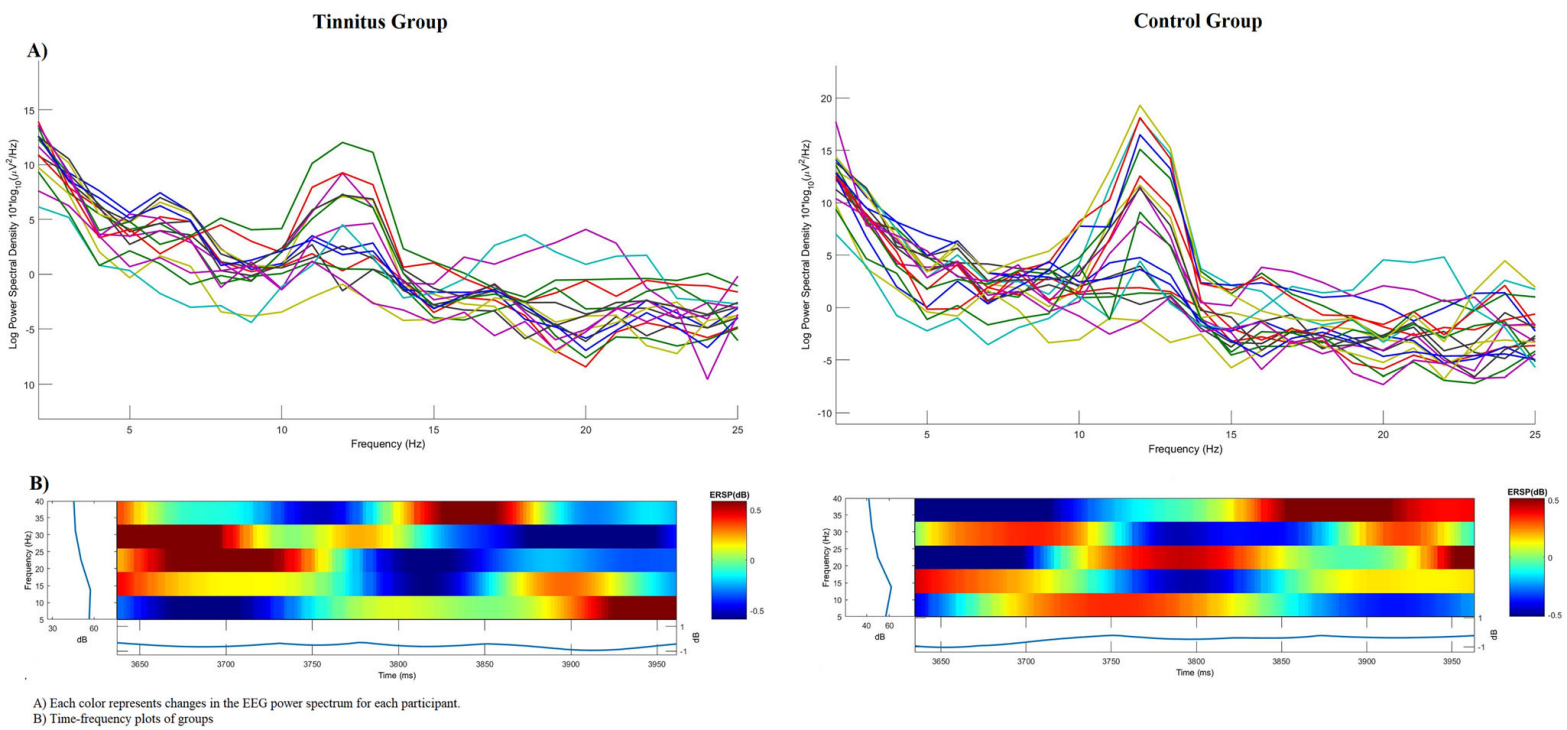
Time‐dependent change of EEG power spectrum according to P*z* electrode and time–frequency plots for each group. EEG, electroencephalography.

The mean EEG alpha band power calculated at baseline was 4.16 ± 2.68 µV for the tinnitus group and 5.03 ± 3.89 µV for the control group. There was no statistically significant difference between the two groups (*p* = 0.41). Table 3 shows the mean EEG alpha power change relative to baseline and statistical evaluation of the groups for the 6‐channel 50% SRT listening situation. The independent sample *t*‐test revealed that the tinnitus group exhibited a comparatively smaller increase in EEG alpha band power (*p* = 0.001). Figure 2 shows the time dependence of the EEG alpha band power change according to the groups. Figure 3 shows the mean EEG alpha power change differences between groups with error bars. Figure 4 shows the time‐dependent change of EEG power spectrum according to Pz electrode and time‐frequency plots for each group.

**TABLE 3:** Mean electroencephalography (EEG) alpha power change differences between the tinnitus (*n* = 16) and control (*n* = 23) groups and statistical evaluation.

Variable	Tinnitus group		Control group		
	Mean (%)	Std.	Mean (%)	Std.	*p* value
EEG alpha power change	105.88	128.64	225.30	66.88	0.003^*^

Abbreviation: Std., standard deviation.

* independent samples *t*‐test, statistically significant difference.

EEG alpha band power change according to RI status could not be evaluated because the number of participants with RI+ and RI− was not evenly distributed. EEG alpha band power change based on the RI status could not be evaluated because the number of participants with RI+ and RI− was not evenly distributed. It was concluded that a statistically robust comparison could not be made.

### 3.3 Investigation of Possible Causes of Changes in EEG Alpha Band Power

#### 3.3.1 Evaluation of the Relationship Between THI and EEG Alpha Band Power

Statistical analysis using Pearson's correlation coefficient revealed no significant correlation between the change in alpha band power of the EEG and the scores of the THI (*p* = 0.71).

### 3.4 Comparison of ABRs Between Groups

**FIGURE 5:**
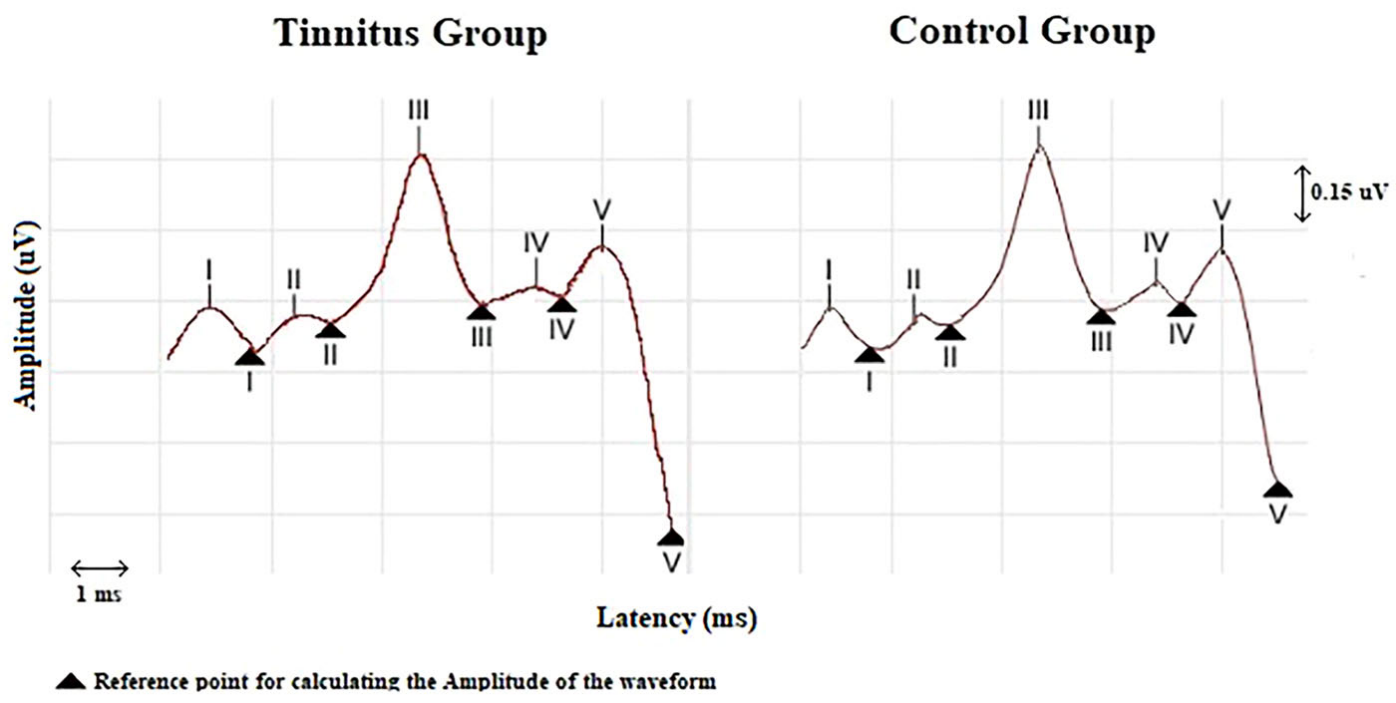
ABR waveforms of the groups. ABR, auditory brainstem response.

**FIGURE 6:**
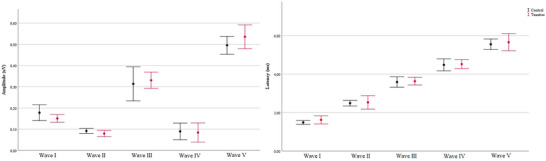
Error bar presentation of amplitude and latency values of ABR waveforms. ABR, auditory brainstem response.

Table 4 shows the amplitude and absolute latency values of the ABR waveforms of the groups. For ABR, a total of 32 ears in the tinnitus group and 46 ears in the control group were evaluated. There was no statistically significant difference between the groups in terms of any ABR component (*p* > 0.05). Furthermore, ABR waveforms of the groups are shown in Figure 5. Amplitude and latency values of ABR waveforms are shown with error bars in Figure 6.

**TABLE 4:** Amplitude and absolute latency values of the groups.

	Amplitude			Absolute latency		
Wave	Tinnitus group	Control group	*p* value	Tinnitus group	Control group	*p* value
	Mean ± Std.	Mean ± Std.		Mean ± Std.	Mean ± Std.	
I	0.15 ± 0.03	0.17 ± 0.08	0.18	1.50 ± 0.24	1.56 ± 0.36	0.55
II	0.07 ± 0.02	0.09 ± 0.02	0.17	2.52 ± 0.65	2.48 ± 0.33	0.80
III	0.33 ± 0.07	0.31 ± 0.18	0.69	3.63 ± 0.37	3.59 ± 0.63	0.79
IV	0.08 ± 0.08	0.08 ± 0.09	0.84	4.52 ± 0.43	4.48 ± 0.72	0.84
V	0.53 ± 0.1	0.49 ± 0.09	0.23	5.66 ± 0.83	5.55 ± 0.63	0.65
III/I	2.28 ± 0.62	2.21 ± 1.64	0.86			
V/I	3.76 ± 1.15	3.64 ± 2.45	0.83			

### 3.5 Comparison of SPIN Test (MT) and VAS Scores Between Groups

Table 5 shows the mean 50% SRT and VAS scores and statistical evaluation of the groups. For MT, a total of 32 ears in the tinnitus group and 46 ears in the control group were evaluated. Using the independent sample *t*‐test, VAS score is higher in tinnitus group (*p* = 0.003).

**TABLE 5:** Mean 50% speech reception thresholds (SRT) scores and statistical evaluation of the groups.

Variable	Tinnitus group	Control group	*p* value
50% SRT	1.63 ± 0.37	1.56 ± 0.38	0.62
VAS	4.93 ± 2.25 (1–8)	2.04 ± 1.58 (0–5)	<0.01^*^

Abbreviations: Std., standard deviation; VAS, visual analogue scale.

* independent samples *t*‐test, statistically significant difference.

## 4 Discussion

The primary goal of this study was to investigate listening effort in tinnitus patients by ensuring as much PAF similarity as possible between the groups. We used ABR and MT to ensure PAF similarity, and there was no difference between the groups for either method. We aimed to assess EEG alpha band power differences in the tinnitus and control groups as a reflection of the CNS to measure listening effort. According to EEG results, the increase in alpha band power was lower in the tinnitus group than in the control group.

### 4.1 EEG Alpha Band Power

Previous studies have shown that changes in the EEG alpha band power are closely related to the allocation of neural resources to a stimulus presented during active listening (Dimitrijevic et al. 2019). This relationship can be explained as follows: in an active listening state, the effort to analyze the presented stimulus increases, and, correspondingly, the number of neural resources allocated for encoding the stimulus in the brain also increases (Klimesch et al. 2007). As more neural resources are allocated for encoding, the inhibition mechanism in the brain is reduced (Miles et al. 2017). Studies have shown that EEG alpha band power is closely linked to the inhibition mechanism in the brain (Miles et al. 2017). Specifically, EEG alpha band power decreases when inhibition decreases, and it increases when inhibition increases (Klimesch et al. 2007). Therefore, with increased effort during the processing of a stimulus, the overall inhibition mechanism in the brain decreases, leading to a decrease in EEG alpha band power (Klimesch et al. 2007).

In a previous study, participants were presented with four different auditory stimuli of varying difficulty levels (Klimesch 2012; Weisz et al. 2011). The results showed that EEG alpha band power increased when participants experienced minimal effort and decreased when effort was highest. However, studies have also shown that once the stimulus difficulty reaches a certain threshold, EEG alpha band power begins to increase again (Li et al. 2020). As the auditory stimulus becomes more challenging, participants are less able to access the acoustic cues required for comprehension (Klimesch 2012). At this point, participants lose interest in the stimulus, and the neural resources used by the brain to decode the auditory stimulus are reduced (Li et al. 2020). This phenomenon is known as listening fatigue (Li et al. 2020).

Thus, the level of difficulty of the stimulus used in listening effort studies is crucial. To address this, we used stimuli that have previously been employed to assess listening effort and have produced consistent results without causing listening fatigue (Miles et al. 2017).

Previous studies have shown that changes in the EEG alpha band power are closely related to the allocation of neural resources to a stimulus presented during active listening (Dimitrijevic et al. 2019). In active listening, the effort to analyze the presented stimulus increases, and, thus, the number of neural resources allocated for encoding the stimulus in the brain also increases (Klimesch et al. 2007). Subsequently, the current inhibition mechanism in the brain decreases due to increased neural resources for encoding (Miles et al. 2017). Studies have shown that the EEG alpha band power is closely linked to the inhibition mechanism in the brain (Miles et al. 2017). Specifically, EEG alpha band power decreases when inhibition decreases, and it increases when inhibition increases (Klimesch et al. 2007). Therefore, with the effort caused by the presented stimulus, the general inhibition mechanism in the brain decreases, and a decrease in the EEG alpha band power can be observed (Klimesch et al. 2007). In a previous study, participants were presented with four different auditory stimuli of varying difficulty (Klimesch 2012; Weisz et al. 2011). As a result, it was shown that the EEG alpha band power increased when the participants’ effort was the least and decreased when the effort was the most. However, studies have shown that once the stimulus difficulty reaches a certain point, the EEG alpha band power begins to increase (Li et al. 2020). As the auditory stimulus becomes more difficult, participants will no longer receive the acoustic cues required to comprehend it (Klimesch 2012). They lose interest in the stimulus at this point, and the neural resources used by the brain to decode the auditory stimulus are reduced (Li et al. 2020). This is commonly referred as listening fatigue (Li et al. 2020). Therefore, the level of difficulty of the stimulus used in listening effort studies is important. Hence, we used stimuli that have previously been used to assess listening effort and have produced consistent results that do not cause listening fatigue (Miles et al. 2017).

### 4.2 Importance of Ensuring Similar PAF Across Groups

The most important difference of this study from previous studies is that the possible effect of PAD on listening effort was considered, having PAF similarity between the groups. It has been shown that tinnitus patients may have PAD even if their pure tone hearing thresholds are normal (Kara et al. 2020; Ahmadpour et al. 2022; Sendesen et al. 2022). Although hearing thresholds between 0.125 and 20 kHz were normal for both groups in the current study, PAD may still affect the temporal processing (Monaghan et al. 2020; Kamerer et al. 2019; Henry 2022). This can impair short‐term memory, working memory, and attention, which are closely related to listening effort (Francis and Love 2020; Degeest, Kestens, and Keppler 2022; Alhanbali et al. 2019; Peelle 2018). Damage to these processes may cause listening difficulties (Kamerer et al. 2019; Henry 2022), although it has not yet been shown in the literature to directly cause listening effort. Therefore, as previously suggested, when evaluating the effect of tinnitus on listening effort, it is very important to ensure that the PAF in this patient group is similar to the control group. Here, we used MT and ABR to indicate PAF similarity in the present study (Bajin, Dahm, and Lin 2022; Plack et al. 2016). There was no difference between the groups in MT scores and ABR components. The lack of difference between the groups in these evaluation methods may be evidence of PAF similarity between the groups.

### 4.3 Auditory Brainstem Responses

In ABR, there was no difference between the groups in I–V waveform absolute latency and amplitude values, as well as III/I and V/I amplitude ratios in current study. In the literature, there are studies indicating that ABR wave amplitudes and ratios differ in tinnitus patients compared to the control group. However, EHF was not evaluated in these studies. As is known, a decrease in high frequency hearing thresholds may cause a decrease in ABR wave amplitudes (Sendesen et al. 2022; Chen et al. 2021). As the etiology of tinnitus is often associated with high frequency hearing loss, it is highly likely that high frequency hearing thresholds are lower in the patient group (Jafari et al. 2022). Therefore, as EHF hearing thresholds were not evaluated in other studies, the etiology of tinnitus may be associated with high frequency hearing loss. However, in this study, the etiology of tinnitus probably differed from these studies as hearing thresholds in the range of 0.125–20 kHz were within normal limits. Therefore, ABRs may have differed in our study compared to previous studies. The difference in ABR waveform between groups in the studies may also be associated with tinnitus etiology. As is known, tinnitus etiology may be related to PAD (Chen et al. 2021; Colak, Sendesen, and Turkyilmaz 2024; Langguth et al. 2013), but it may also be related to non‐PAF etiologies such as somatosensory tinnitus (Møller and De Ridder 2024). When tinnitus etiology is related to PAD, amplitude changes (decrease in Wave I, increase in Wave III and/or Wave V) can sometimes be seen in ABR waveforms (Bajin, Dahm, and Lin 2022; Plack et al. 2016; Lobarinas, Spankovich, and Le Prell 2017). However, when PAF is not affected, no effect may be seen in ABR waveforms that provide information about the auditory nerve and the caudal part of the brainstem (Makar 2021; Santiago, Romão, and Gil 2023). Therefore, unlike previous studies, the present study may have included participants with different non‐PAF tinnitus etiologies. Therefore, no difference may have been obtained between groups in terms of ABR waveforms. There are also different studies in the literature, in which no difference was observed between the groups in terms of ABR waveforms (Turner et al. 2022; Milloy et al. 2017).

Considering the ABR findings obtained from this study, PAF can be interpreted as similar between the groups. Therefore, the listening effort in tinnitus may be related to central dysfunction rather than PAD caused by tinnitus.

### 4.4 SPIN Test

Another noteworthy finding of this study was that, although there was no difference between the groups in terms of MT, findings indicating increased listening effort were obtained in the tinnitus group based on the EEG alpha band and VAS scores. This shows that SPIN tests, such as MT, do not provide enough information about how much effort individuals exert while listening. This may indicate that listening difficulties, such as increased listening effort, could be present in individuals who present to the clinic with complaints related to SPIN but have normal results in tests evaluating SPIN ability. Thus, some individuals may have experience listening difficulties despite normal SPIN performance. Previous studies have also shown differences in listening effort between these groups, although there was no difference between the groups in the SPIN test (Sendesen and Turkyilmaz 2024; Sendesen and Türkyılmaz 2024; Gosselin and Gagné 2011; Sendesen et al. 2023). The reason for this may be that although SPIN ability is related to central auditory system, it is also closely related to the early stages of the peripheral and central auditory system (Yeend et al. 2017; Lazard, Collette, and Perrot 2012), while listening effort assessment methods are predominantly related to central auditory and nonauditory areas related to neural resource management in the brain (Alhanbali et al. 2019; Amichetti et al. 2013; Rudner 2016). In conclusion, these findings suggest that for patients who report difficulty listening in noise, the focus should not only be on SPIN performance but also on the effort these patients may be exerting to listen.

### 4.5 Possible Reasons for the Effect of Tinnitus on Listening Effort

Previous work suggested that the reason for the listening effort in tinnitus patients was that their attention was focused on tinnitus rather than on the auditory stimulus presented (Degeest, Keppler, and Corthals 2017). However, if attention shifts to the tinnitus instead of the auditory stimulus, the number of neural resources allocated for encoding the auditory stimulus would decrease, leading to an expectation of higher EEG alpha band power during stimulus encoding compared to the control group. There is evidence that the EEG alpha band power increases with cortical inhibition (Klimesch et al. 2007). In addition, considering that the auditory stimulus level was 65 dB SPL, many participants may not have perceived their tinnitus, as their MMLs were below 65 dB. Therefore, it is less likely that participants were distracted by tinnitus during the assessment. We also argue that this may provide evidence that increased listening effort is unrelated to distraction caused by tinnitus perception and is instead relevant to the pathophysiological mechanism of tinnitus.

Attention plays an active role in the listening situations, as it is an important cognitive indicator (Worthington 2017). Previous studies have shown that attention is weaker in tinnitus patients (Dornhoffer et al. 2006; Stevens et al. 2007; Maloney, Sattizahn, and Beilock 2014). However, if the participants’ attention had been diverted from the auditory stimulus, an increase in EEG alpha band power would be expected, reflecting fewer neural resources used to analyze the auditory stimulus. Therefore, on the basis of changes in EEG alpha band power, it is possible to infer that participants in our study did not direct their attention away from the stimulus. Furthermore, considering that attention is also a cognitive skill, there was no difference between the groups in MoCA scores, which provide basic information about cognitive skills. This suggests that there is no fundamental difference between the groups in terms of attention. However, as the MoCA is not a method that directly evaluates attention skills, the results should be interpreted with caution. All these factors may indicate that the primary cause of increased listening effort in tinnitus patients is not due to diverted attention from the auditory stimulus.

Prior research has demonstrated that tinnitus can induce chronic anxiety in patients (Cho et al. 2013). Tinnitus patients may experience an impairment in the utilization of cognitive resources due to anxiety, which may manifest as increased effort during listening (Maloney, Sattizahn, and Beilock 2014; Butler and Mathews 1983; Uygur and Arslan 2010; Grant and White 2016). Therefore, the intensity of anxiety that tinnitus patients experience in their daily lives was assessed using the THI in this study. However, the current study found no correlation between THI scores and the changes in EEG alpha band power. This result suggests that there may not be a relationship between listening effort and the anxiety that participants experience due to their tinnitus in daily life.

Motivation, on the other hand, is one of the listener‐dependent factors that can affect listening effort. To minimize motivational variation between participants, all assessments were conducted by a single expert who followed the same instructions for all participants. Although we could not fully equalize motivation across participants, we aimed to reduce its effect by avoiding differences in expert assessments or instructions that could lead to motivational variation.

The main difference of this study compared to other studies is that the hearing thresholds in the range of 0.125–20 kHz did not differ between the groups, allowing the effect of tinnitus on listening effort to be evaluated in isolation as much as possible by providing similar PAF between the groups. Although previous research supports the presence of increased listening effort in tinnitus patients, the pathophysiologic basis of tinnitus imposes limitations. One model explaining the pathophysiology of tinnitus is the thalamocortical dysrhythmia model. According to this model, the inhibitory effect of cortical pathways on peripheral auditory pathways begins to diminish due to the lack of input from peripheral auditory pathways, resulting in hyperexcitation of the central auditory pathways (Weisz, Dohrmann, and Elbert 2007). Studies have shown that EEG alpha band power is directly related to the level of cortical inhibition (Klimesch et al. 2007; Kostandyan et al. 2019; Seifi Ala et al. 2020). In previous studies investigating the effect of tinnitus on listening effort with EEG, although hearing thresholds were symmetrical between groups, hearing loss was present above 8 kHz (Sendesen and Turkyilmaz 2024; Sendesen et al. 2023). This may have caused a lack of peripheral auditory input, contributing to the increase in EEG alpha band power interpreted as increased listening effort, in line with the thalamocortical dysrhythmia model. In another study investigating the effect of tinnitus on listening effort using pupillometry, the hearing thresholds of both the tinnitus group and the control group were within normal limits in the range of 0.125–20 kHz (Sendesen and Türkyılmaz 2024). However, it is known that the normal hearing thresholds of the groups do not rule out PAD. Considering that a possible imbalance of peripheral auditory input between groups may cause listening difficulties and changes in EEG alpha power independent of listening effort, as suggested by the thalamocortical dysrhythmia model, we suggest that it is important to ensure similar PAF between the groups using ABR and MT in the present study.

### 4.6 Limitations

The current study has several limitations. Here, in this study, we discussed the potential causes of increased listening effort in tinnitus patients based on the findings from previous research. Future research should consider including methods that can assess the potential cause of increased listening effort. Moreover, this study was a single‐center study, and we were able to recruit patients who presented to our clinic within a limited time frame. In such circumstances, participant diversity may be limited, potentially introducing recruitment bias. In this study, we used ABR and MT to ensure PAF similarity between the groups. To our knowledge, there is currently no other method to clearly demonstrate PAD other than histopathological investigation methods. Therefore, we think that the results of our study should be interpreted with caution. In the current study, VAS was one of the methods we used to assess the listening effort of the participants. However, it should be noted that this method is not a standardized method. Furthermore, the sample size of our study is relatively small, which should be considered when interpreting the results. Although the stimuli presented in the present study are based on previous research, individual characteristics of the participants (such as age, education, cultural differences, and vocabulary) may have led some participants to exert greater effort than others, potentially even causing fatigue. Future studies may consider using standardized stimuli, if available, and creating more homogeneous study groups.

## 5 Conclusion

To the best of our knowledge, this study represents the first attempt to investigate listening effort based on EEG alpha‐band power while maintaining normal hearing thresholds of 0.125–20 kHz between groups and ensuring PAF similarity. The lower increase in EEG alpha‐band power in the tinnitus group may be evidence of increased listening effort in tinnitus patients. The primary goal of clinicians in tinnitus patients is generally to improve and relieve the tinnitus perception. However, as observed in our study, tinnitus also has cognitive effects, such as listening problems. Therefore, clinicians may be encouraged to add outcomes for improving listening skills to their treatment/therapy programs for tinnitus perception. In summary, despite maintaining comparable PAF across groups, the tinnitus group showed a greater demand for effort during listening. Future studies could investigate the effect of tinnitus on listening effort in the central system rather than the peripheral system.

## Author Contributions

**Eser Sendesen**: conceptualization, investigation, writing–original draft, writing–review and editing, visualization, methodology, data curation, resources. **Meral Didem Turkyilmaz**: Supervision, project administration.

## Conflicts of Interest

The authors declare no conflicts of interest.

### Peer Review

The peer review history for this article is available at https://publons.com/publon/10.1002/brb3.70306.

## Data Availability

The data that support the findings of this study are available from the corresponding author (Eser Sendesen), upon request.

## References

[brb370306-bib-0001] Ahmadpour, T. , R. Toufan , A. Pourbakht , and M. Kamali . 2022. “Evaluation of Cochlear Synaptopathy in Tinnitus Patients With Normal Hearing Using Auditory Brainstem Response and Electrocochleography Tests.” Auditory and Vestibular Research 31, no. 1: 4–10.

[brb370306-bib-0002] Aksoy, S. , Y. Firat , and R. Alpar . 2007. “The Tinnitus Handicap Inventory: A Study of Validity and Reliability.” International Tinnitus Journal 13, no. 2: 94–98. 18229787

[brb370306-bib-0003] Alhanbali, S. , P. Dawes , R. E. Millman , and K. J. Munro . 2019. “Measures of Listening Effort Are Multidimensional.” Ear and Hearing 40, no. 5: 1084–1097. 30747742 10.1097/AUD.0000000000000697 PMC7664710

[brb370306-bib-0076] Altın, B. , G. İ. Şahin Kamışlı , and S. Aksoy . 2023. “Validity and Reliability Study of the Khalfa's Hyperacusis Questionnaire with Using ULL in Tinnitus Patients.” European Archives of Oto‐Rhino‐Laryngology 280, no. 3: 1485–1492. 36334111 10.1007/s00405-022-07727-7

[brb370306-bib-0004] Amichetti, N. M. , R. S. Stanley , A. G. White , and A. Wingfield . 2013. “Monitoring the Capacity of Working Memory: Executive Control and Effects of Listening Effort.” Memory & Cognition 41, no. 6: 839–849. 23400826 10.3758/s13421-013-0302-0 PMC3718871

[brb370306-bib-0005] Bajin, M. D. , V. Dahm , and V. Y. Lin . 2022. “Hidden Hearing Loss: Current Concepts.” Current Opinion in Otolaryngology & Head and Neck Surgery 30, no. 5: 321–325. 36004790 10.1097/MOO.0000000000000824

[brb370306-bib-0006] Basile, C.‐É. , P. Fournier , S. Hutchins , and S. Hébert . 2013. “Psychoacoustic Assessment to Improve Tinnitus Diagnosis.” PLoS ONE 8, no. 12: e82995. 24349414 10.1371/journal.pone.0082995 PMC3861445

[brb370306-bib-0007] Butler, G. , and A. Mathews . 1983. “Cognitive Processes in Anxiety.” Advances in Behaviour Research and Therapy 5, no. 1: 51–62.

[brb370306-bib-0008] Juul Jensen, J. , S. L. Callaway , T. Lunner , and D. Wendt . 2018. “Measuring the Impact of Tinnitus on Aided Listening Effort Using Pupillary Response.” Trends in Hearing 22: 2331216518795340. 10.1177/2331216518795340. 30205768 PMC6136111

[brb370306-bib-0009] Cartocci, G. , B. M. S. Inguscio , G. Giliberto , et al. 2023. “Listening Effort in Tinnitus: A Pilot Study Employing a Light EEG Headset and Skin Conductance Assessment During the Listening to a Continuous Speech Stimulus Under Different SNR Conditions.” Brain Sciences 13, no. 7: 1084. 37509014 10.3390/brainsci13071084 PMC10377270

[brb370306-bib-0010] Chen, F. , F. Zhao , N. Mahafza , and W. Lu . 2021. “Detecting Noise‐Induced Cochlear Synaptopathy by Auditory Brainstem Response in Tinnitus Patients With Normal Hearing Thresholds: A Meta‐Analysis.” Frontiers in Neuroscience 15: 778197. 34987358 10.3389/fnins.2021.778197 PMC8721093

[brb370306-bib-0011] Cho, C. G. , J. H. Chi , J.‐J. Song , E. K. Lee , and B. H. Kim . 2013. “Evaluation of Anxiety and Depressive Levels in Tinnitus Patients.” Korean Journal of Audiology 17, no. 2: 83–89. 24653912 10.7874/kja.2013.17.2.83 PMC3936536

[brb370306-bib-0012] Çolak, H. , B. E. Aydemir , M. D. Sakarya , E. Çakmak , A. Alniaçik , and M. D. Türkyilmaz . 2024. “Subcortical Auditory Processing and Speech Perception in Noise Among Individuals With and Without Extended High‐Frequency Hearing Loss.” Journal of Speech, Language, and Hearing Research 67, no. 1: 221–231. 10.1044/2023_JSLHR-23-00023 37956878

[brb370306-bib-0013] Colak, H. , E. Sendesen , and M. D. Turkyilmaz . 2024. “Subcortical Auditory System in Tinnitus With Normal Hearing: Insights From Electrophysiological Perspective.” European Archives of Oto‐Rhino‐Laryngology 281: 4133–4142. 10.1007/s00405-024-08583-3. 38555317 PMC11266230

[brb370306-bib-0014] Dalrymple‐Alford, J. , M. MacAskill , C. Nakas , et al. 2010. “The MoCA: Well‐Suited Screen for Cognitive Impairment in Parkinson Disease.” Neurology 75, no. 19: 1717–1725. 21060094 10.1212/WNL.0b013e3181fc29c9

[brb370306-bib-0015] Degeest, S. , H. Keppler , and P. Corthals . 2017. “The Effect of Tinnitus on Listening Effort in Normal‐Hearing Young Adults: A Preliminary Study.” Journal of Speech, Language, and Hearing Research 60, no. 4: 1036–1045. 10.1044/2016_JSLHR-H-16-0090 28282482

[brb370306-bib-0016] Degeest, S. , K. Kestens , and H. Keppler . 2022. “Investigation of the Relation Between Tinnitus, Cognition, and the Amount of Listening Effort.” Journal of Speech, Language, and Hearing Research 65, no. 5: 1988–2002. 10.1044/2022_JSLHR-21-00347 35377707

[brb370306-bib-0017] Dillon, H. , and S. Cameron . 2021. “Separating the Causes of Listening Difficulties in Children.” Ear and Hearing 42, no. 5: 1097–1108. 34241982 10.1097/AUD.0000000000001069 PMC8378540

[brb370306-bib-0018] Dimitrijevic, A. , M. L. Smith , D. S. Kadis , and D. R. Moore . 2019. “Neural Indices of Listening Effort in Noisy Environments.” Scientific Reports 9, no. 1: 1–10. 31375712 10.1038/s41598-019-47643-1 PMC6677804

[brb370306-bib-0019] Dornhoffer, J. , C. Danner , M. Mennemeier , D. Blake , and E. Garcia‐Rill . 2006. “Arousal and Attention Deficits in Patients With Tinnitus.” International Tinnitus Journal 12, no. 1: 9–16. 17147035

[brb370306-bib-0020] Francis, A. L. , and J. Love . 2020. “Listening Effort: Are We Measuring Cognition or Affect, or Both?” Wiley Interdisciplinary Reviews: Cognitive Science 11, no. 1: e1514. 31381275 10.1002/wcs.1514

[brb370306-bib-0021] Galazyuk, A. , R. Longenecker , S. Voytenko , I. Kristaponyte , and G. Nelson . 2019. “Residual Inhibition: From the Putative Mechanisms to Potential Tinnitus Treatment.” Hearing Research 375: 1–13. 30822633 10.1016/j.heares.2019.01.022

[brb370306-bib-0022] Giuliani, N. P. , C. J. Brown , and Y.‐H. Wu . 2021. “Comparisons of the Sensitivity and Reliability of Multiple Measures of Listening Effort.” Ear and Hearing 42, no. 2: 465–474. 32925306 10.1097/AUD.0000000000000950 PMC9135174

[brb370306-bib-0023] Gosselin, P. A. , and J.‐P. Gagné . 2011. “Older Adults Expend More Listening Effort Than Young Adults Recognizing Speech in Noise.” Journal of Speech, Language, and Hearing Research 54: 944–958. 10.1044/1092-4388(2010/10-0069) 21060138

[brb370306-bib-0024] Grant, D. M. , and E. J. White . 2016. “Influence of Anxiety on Cognitive Control Processes.” In Oxford Research Encyclopedia of Psychology. Oxford University Press.

[brb370306-bib-0025] Gürses, E. , S. Ercan , M. D. Türkyılmaz , and S. Aksoy . 2018. “Tinnituslu bireylerde dinleme eforunun değerlendirilmesi: Bir ön çalışma.” Türk Odyoloji Ve İşitme Araştırmaları Dergisi 1, no. 1: 15–20.

[brb370306-bib-0026] Henry, K. S. 2022. “Animal Models of Hidden Hearing Loss: Does Auditory‐Nerve‐Fiber Loss Cause Real‐World Listening Difficulties?” Molecular and Cellular Neuroscience 118: 103692. 10.1016/j.mcn.2021.103692. 34883241 PMC8928575

[brb370306-bib-0027] Huang, C.‐Y. , D.‐S. Li , M.‐H. Tsai , C.‐H. Chen , and Y.‐F. Cheng . 2022. “The Impact of Acute Tinnitus on Listening Effort: A Study Based on Clinical Observations of Sudden Sensorineural Hearing Loss Patients.” International Journal of Environmental Research and Public Health 19, no. 6: 3661. 10.3390/ijerph19063661. 35329346 PMC8955353

[brb370306-bib-0028] Jafari, Z. , D. Baguley , B. E. Kolb , and M. H. Mohajerani . 2022. “A Systematic Review and Meta‐Analysis of Extended High‐Frequency Hearing Thresholds in Tinnitus With a Normal Audiogram.” Ear and Hearing 43, no. 6: 1643–1652. 35612517 10.1097/AUD.0000000000001229

[brb370306-bib-0029] Jensen, M. , E. Hüttenrauch , J. Müller‐Mazzotta , B. A. Stuck , and C. Weise . 2021. “On the Impairment of Executive Control of Attention in Chronic Tinnitus: Evidence From the Attention Network Test.” Behavioural Brain Research 414: 113493. 34329668 10.1016/j.bbr.2021.113493

[brb370306-bib-0030] Kamerer, A. M. , A. AuBuchon , S. E. Fultz , J. G. Kopun , S. T. Neely , and D. M. Rasetshwane . 2019. “The Role of Cognition in Common Measures of Peripheral Synaptopathy and Hidden Hearing Loss.” American Journal of Audiology 28, no. 4: 843–856. 31647880 10.1044/2019_AJA-19-0063 PMC7210438

[brb370306-bib-0031] Kara, E. , K. Aydın , A. A. Akbulut , et al. 2020. “Assessment of Hidden Hearing Loss in Normal Hearing Individuals With and Without Tinnitus.” Journal of International Advanced Otology 16, no. 1: 87–92. 32209515 10.5152/iao.2020.7062 PMC7224424

[brb370306-bib-0077] Kılıç, S. , E. Sendesen , F. Aslan , N. Erbil , Ö. Aydın , and D. Türkyılmaz . 2024. “Investigating Sensitivity to Auditory Cognition in Listening Effort Assessments: A Simultaneous EEG and Pupillometry Study.” Brain and Behavior 14, no. 11: e70135. 39482842 10.1002/brb3.70135 PMC11527829

[brb370306-bib-0032] Kim, Y. , and W. Han . 2023. “Recommended Auditory Brainstem Responses to Stimulation and Recording Parameters for Hidden Hearing Loss: A Systematic Review and Meta‐Analysis.” Audiology and Speech Research 19, no. 2: 77–90.

[brb370306-bib-0033] Klimesch, W. 2012. “Alpha‐Band Oscillations, Attention, and Controlled Access to Stored Information.” Trends in Cognitive Sciences 16, no. 12: 606–617. 23141428 10.1016/j.tics.2012.10.007 PMC3507158

[brb370306-bib-0034] Klimesch, W. , P. Sauseng , and S. Hanslmayr . 2007. “EEG Alpha Oscillations: The Inhibition–Timing Hypothesis.” Brain Research Reviews 53, no. 1: 63–88. 16887192 10.1016/j.brainresrev.2006.06.003

[brb370306-bib-0035] Kostandyan, M. , K. Bombeke , T. Carsten , R. M. Krebs , W. Notebaert , and C. N. Boehler . 2019. “Differential Effects of Sustained and Transient Effort Triggered by Reward – A Combined EEG and Pupillometry Study.” Neuropsychologia 123: 116–130. 29709582 10.1016/j.neuropsychologia.2018.04.032

[brb370306-bib-0036] Langguth, B. , P. M. Kreuzer , T. Kleinjung , and D. De Ridder . 2013. “Tinnitus: Causes and Clinical Management.” Lancet Neurology 12, no. 9: 920–930. 23948178 10.1016/S1474-4422(13)70160-1

[brb370306-bib-0037] Lazard, D. S. , J. L. Collette , and X. Perrot . 2012. “Speech Processing: From Peripheral to Hemispheric Asymmetry of the Auditory System.” Laryngoscope 122, no. 1: 167–173. 22095864 10.1002/lary.22370

[brb370306-bib-0038] Li, G. , S. Huang , W. Xu , et al. 2020. “The Impact of Mental Fatigue on Brain Activity: A Comparative Study Both in Resting State and Task State Using EEG.” BMC Neuroscience 21: 1–9. 32398004 10.1186/s12868-020-00569-1 PMC7216620

[brb370306-bib-0039] Lobarinas, E. , C. Spankovich , and C. G. Le Prell . 2017. “Evidence of “Hidden Hearing Loss” Following Noise Exposures That Produce Robust TTS and ABR Wave‐I Amplitude Reductions.” Hearing Research 349: 155–163. 10.1016/j.heares.2016.12.009. 28003148

[brb370306-bib-0040] Makar, S. K. 2021. “Etiology and Pathophysiology of Tinnitus: A Systematic Review.” International Tinnitus Journal 25, no. 1: 76–86. 34410084 10.5935/0946-5448.20210015

[brb370306-bib-0041] Maloney, E. A. , J. R. Sattizahn , and S. L. Beilock . 2014. “Anxiety and Cognition.” Wiley Interdisciplinary Reviews: Cognitive Science 5, no. 4: 403–411. 26308653 10.1002/wcs.1299

[brb370306-bib-0042] Miles, K. , C. McMahon , I. Boisvert , et al. 2017. “Objective Assessment of Listening Effort: Coregistration of Pupillometry and EEG.” Trends in Hearing 21: 2331216517706396. 28752807 10.1177/2331216517706396 PMC5536372

[brb370306-bib-0043] Milloy, V. , P. Fournier , D. Benoit , A. Noreña , and A. Koravand . 2017. “Auditory Brainstem Responses in Tinnitus: A Review of Who, How, and What?” Frontiers in Aging Neuroscience 9: 237. 28785218 10.3389/fnagi.2017.00237 PMC5519563

[brb370306-bib-0044] Møller, A. R. , and D. De Ridder . 2024. “Tinnitus and the Somatosensory System.” In Textbook of Tinnitus, 135–143. Springer.

[brb370306-bib-0045] Monaghan, J. J. , J. A. Garcia‐Lazaro , D. McAlpine , and R. Schaette . 2020. “Hidden Hearing Loss Impacts the Neural Representation of Speech in Background Noise.” Current Biology 30, no. 23: 4710–4721.e4. 33035490 10.1016/j.cub.2020.09.046 PMC7728162

[brb370306-bib-0046] Neagu, M.‐B. , A. A. Kressner , H. Relaño‐Iborra , P. Bækgaard , T. Dau , and D. Wendt . 2023. “Investigating the Reliability of Pupillometry as a Measure of Individualized Listening Effort.” Trends in Hearing 27: 23312165231153288.

[brb370306-bib-0047] Obleser, J. , and N. Weisz . 2012. “Suppressed Alpha Oscillations Predict Intelligibility of Speech and Its Acoustic Details.” Cerebral Cortex 22, no. 11: 2466–2477. 22100354 10.1093/cercor/bhr325 PMC4705336

[brb370306-bib-0048] Peelle, J. E. 2018. “Listening Effort: How the Cognitive Consequences of Acoustic Challenge Are Reflected in Brain and Behavior.” Ear and Hearing 39, no. 2: 204–214. 28938250 10.1097/AUD.0000000000000494 PMC5821557

[brb370306-bib-0049] Pichora‐Fuller, M. K. , S. E. Kramer , M. A. Eckert , et al. 2016. “Hearing Impairment and Cognitive Energy: The Framework for Understanding Effortful Listening (FUEL).” Ear and Hearing 37: 5S–27S. 27355771 10.1097/AUD.0000000000000312

[brb370306-bib-0050] Pienkowski, M. 2017. “On the Etiology of Listening Difficulties in Noise Despite Clinically Normal Audiograms.” Ear and Hearing 38, no. 2: 135–148. 28002080 10.1097/AUD.0000000000000388 PMC5325255

[brb370306-bib-0051] Plack, C. J. , A. Léger , G. Prendergast , K. Kluk , H. Guest , and K. J. Munro . 2016. “Toward a Diagnostic Test for Hidden Hearing Loss.” Trends in Hearing 20: 2331216516657466. 10.1177/2331216516657466. 27604783 PMC5017571

[brb370306-bib-0052] Rademaker, M. M. , S. M. Meijers , A. L. Smit , and I. Stegeman . 2023. “Prediction Models for Tinnitus Presence and the Impact of Tinnitus on Daily Life: A Systematic Review.” Journal of Clinical Medicine 12, no. 2: 695. 36675624 10.3390/jcm12020695 PMC9861218

[brb370306-bib-0053] Rudner, M. 2016. “Cognitive Spare Capacity as an Index of Listening Effort.” Ear and Hearing 37: 69S–76S. 27355773 10.1097/AUD.0000000000000302

[brb370306-bib-0054] Santiago, J. M. , E. C. Romão , and D. Gil . 2023. “Masking Level Difference and Auditory Brainstem Response in Normal Hearing Adults With Tinnitus.” Distúrbios da Comunicação 35, no. 1: e57675.

[brb370306-bib-0055] Seifi Ala, T. 2022. Evaluating Listening Effort With Electroencephalography in Ecological Situations. University of Nottingham.

[brb370306-bib-0056] Seifi Ala, T. , C. Graversen , D. Wendt , E. Alickovic , W. M. Whitmer , and T. Lunner . 2020. “An Exploratory Study of EEG Alpha Oscillation and Pupil Dilation in Hearing‐Aid Users During Effortful Listening to Continuous Speech.” PLoS ONE 15, no. 7: e0235782. 32649733 10.1371/journal.pone.0235782 PMC7351195

[brb370306-bib-0057] Sendesen, E. , B. Kaynakoglu , L. B. Veziroglu , and M. D. Türkyılmaz . 2022. “Auditory Brainstem Response in Unilateral Tinnitus Patients: Does Symmetrical Hearing Thresholds and Within‐Subject Comparison Affect Responses?” European Archives of Oto‐Rhino‐Laryngology 279, no. 10: 4687–4693. 35098332 10.1007/s00405-021-07232-3

[brb370306-bib-0058] Sendesen, E. , S. Kılıç , N. Erbil , Ö. Aydın , and D. Turkyilmaz . 2023. “An Exploratory Study of the Effect of Tinnitus on Listening Effort Using EEG and Pupillometry.” Otolaryngology–Head and Neck Surgery 169, no. 5: 1259–1267. 37172313 10.1002/ohn.367

[brb370306-bib-0059] Sendesen, E. , and D. Turkyilmaz . 2024. “Investigation of the Behavior of Tinnitus Patients Under Varying Listening Conditions With Simultaneous Electroencephalography and Pupillometry.” Brain and Behavior 14, no. 6: e3571. 38841736 10.1002/brb3.3571 PMC11154813

[brb370306-bib-0060] Sendesen, E. , and M. D. Türkyılmaz . 2024. “Listening Handicap in Tinnitus Patients With Normal Extended High Frequencies From the Perspective of the Autonomic Nervous System–Effort or Fatigue?” Auris, Nasus, Larynx 51, no. 4: 659–665. 38704893 10.1016/j.anl.2024.04.009

[brb370306-bib-0061] Shetty, H. N. , and S. Raju . 2023. “Objective Measure of Listening Effort in HearingImpaired Individuals With and Without Tinnitus.” Journal of International Advanced Otology 19, no. 4: 295–302. 10.5152/iao.2023.22827. 37528594 PMC10543716

[brb370306-bib-0062] Stevens, C. , G. Walker , M. Boyer , and M. Gallagher . 2007. “Severe Tinnitus and Its Effect on Selective and Divided Attention: Acufeno Severo y Sus Efectos sobre la Atención Selectiva y Dividida.” International Journal of Audiology 46, no. 5: 208–216. 17487668 10.1080/14992020601102329

[brb370306-bib-0063] Tietze, G. , and C. Pantev . 1986. “Comparison Between Auditory Brain Stem Responses Evoked by Rarefaction and Condensation Step Functions and Clicks.” Audiology 25, no. 1: 44–53. 3954683 10.3109/00206098609078368

[brb370306-bib-0064] Turner, K. , O. Moshtaghi , N. Saez , et al. 2022. “Auditory Brainstem Response Wave I Amplitude Has Limited Clinical Utility in Diagnosing Tinnitus in Humans.” Brain Sciences 12, no. 2: 142. 35203907 10.3390/brainsci12020142 PMC8870703

[brb370306-bib-0065] Tyler, R. S. , and D. Conrad‐Armes . 1983. “The Determination of Tinnitus Loudness Considering the Effects of Recruitment.” Journal of Speech, Language, and Hearing Research 26, no. 1: 59–72. 10.1044/jshr.2601.59 6865383

[brb370306-bib-0066] Tyler, R. S. , and D. Conrad‐Armes . 1983. “Tinnitus Pitch: A Comparison of Three Measurement Methods.” British Journal of Audiology 17, no. 2: 101–107. 6626779 10.3109/03005368309078916

[brb370306-bib-0067] Uygur, E. , and M. Arslan . 2010. “Effects of Chronic Stress on Cognitive Functions and Anxiety Related Behaviors in Rats.” Acta Physiologica Hungarica 97, no. 3: 297–306. 20843768 10.1556/APhysiol.97.2010.3.6

[brb370306-bib-0068] Waechter, S. , W. J. Wilson , and J. K. Brännström . 2021. “The Impact of Tinnitus on Working Memory Capacity.” International Journal of Audiology 60, no. 4: 274–281. 33000654 10.1080/14992027.2020.1822550

[brb370306-bib-0069] Weisz, N. , K. Dohrmann , and T. Elbert . 2007. “The Relevance of Spontaneous Activity for the Coding of the Tinnitus Sensation.” Progress in Brain Research 166: 61–70. 17956772 10.1016/S0079-6123(07)66006-3

[brb370306-bib-0070] Weisz, N. , T. Hartmann , N. Müller , I. Lorenz , and J. Obleser . 2011. “Alpha Rhythms in Audition: Cognitive and Clinical Perspectives.” Frontiers in Psychology 2: 73. 21687444 10.3389/fpsyg.2011.00073 PMC3110491

[brb370306-bib-0071] Wilson, R. H. , and R. McArdle . 2005. “Speech Signals Used to Evaluate Functional Status of the Auditory System.” Journal of Rehabilitation Research & Development 42: 79–94. 16470466 10.1682/jrrd.2005.06.0096

[brb370306-bib-0072] Wisniewski, M. G. , E. R. Thompson , and N. Iyer . 2017. “Theta‐and Alpha‐Power Enhancements in the Electroencephalogram as an Auditory Delayed Match‐to‐Sample Task Becomes Impossibly Difficult.” Psychophysiology 54, no. 12: 1916–1928. 28792606 10.1111/psyp.12968

[brb370306-bib-0073] Worthington, D. L. 2017. “Modeling and Measuring Cognitive Components of Listening.” In The Sourcebook of Listening Research: Methodology and Measures, 70–96. Wiley.

[brb370306-bib-0074] Yeend, I. , E. F. Beach , M. Sharma , and H. Dillon . 2017. “The Effects of Noise Exposure and Musical Training on Suprathreshold Auditory Processing and Speech Perception in Noise.” Hearing Research 353: 224–236. 28780178 10.1016/j.heares.2017.07.006

[brb370306-bib-0075] Zokoll, M. A. , S. Hochmuth , A. Warzybok , K. C. Wagener , M. Buschermöhle , and B. Kollmeier . 2013. “Speech‐in‐Noise Tests for Multilingual Hearing Screening and Diagnostics1.” American Journal of Audiology 22: 175–178. 23800814 10.1044/1059-0889(2013/12-0061)

